# Exploring the determinants of health service utilization among people living with HIV: a qualitative study in Iran

**DOI:** 10.1186/s12913-023-10321-0

**Published:** 2023-12-04

**Authors:** Neda SoleimanvandiAzar, Salah Eddin Karimi, Sina Ahmadi, Seyed Fahim Irandoost, Ali Amirkafi, Amir Azimi

**Affiliations:** 1 Preventive Medicine and Public Health Research Center, Psychosocial Health Research Institute, Iran University of Medical Sciences, Tehran, Iran; 2 Social Determinants of Health Research Center, Health Management and Safety Promotion Research Institute, Tabriz University of Medical Sciences, Tabriz, Iran; 3 Social Development & Health Promotion Research Center, Health Institute, Kermanshah University of Medical Sciences, Kermanshah, Iran; 4 Department of Community Medicine, School of Medicine, Urmia University of Medical Sciences, Urmia, Iran; 5 Department of Medicine, Iran University of Medical Sciences, Tehran, Iran

**Keywords:** Service utilization, HIV, AIDS, Health, Qualitative study, Iran

## Abstract

**Background:**

Health service utilization among people living with HIV is vital for their survival and quality of life. This study aims to exploring the determinants influencing health service utilization among people living with HIV.

**Methods:**

We conducted a qualitative study involving 16 men and women aged 18–64 living with HIV in Tehran. Data were collected between September and December 2021 through semi-structured interviews conducted via telephone and online platforms, utilizing the purposeful sampling method. Data were analyzed by MAXQDA-2018 software using conventional content analysis approaches and the Granheim and Landman method.

**Results:**

Two main themes, seven categories, and 21 subcategories were obtained from the interviews. The main themes included facilitators of health service utilization (positive personality traits, social factors, and structural-behavioral determinants) and inhibitors of health service utilization (personal conditions, insufficient knowledge and understanding of the disease, negative consequences of disease disclosure, and difficult access to services).

**Conclusion:**

This study underscores the need to invest and expand specialized services for people living with HIV by policy makers, while simultaneously increasing public awareness to reduce the social stigma.

## Introduction

Health care systems are responsible for the health of societies. This responsibility is carried out by providing health care and promotion of the health service utilization (HSU). HSU is defined as the process of using health care services that are available and accessible within a community [1]. Utilization of healthcare services is influenced by many factors that operate at individual, healthcare provider, and social levels [2]. Although HSU is necessary for all members of the society, certain groups need particular attention due to their conditions. People living with HIV (PLHIV) represent one such group, and timely and appropriate access to healthcare services is essential for reducing the viral load, hindering transmission, and preventing the Acquired Immunodeficiency Syndrome (AIDS) phase [3]. AIDS stands as one of the most severe infectious pandemics of the late 20th and early 21st centuries and ranks as the fourth leading cause of death worldwide [4, 5]. As of 2022, about 39 million people globally were living with HIV, with 1.3 million new infections and 630,000 AIDS-related deaths reported [6]. The prevalence of HIV in Iran among the general population is less than 0.01%, but it rises to 17.25% among people who inject drugs [7, 8]. According to the report of Iranian National Electronic Data Management System in 2019, the number of identified PLHIV in Iran and the number of deaths due to AIDS were 41,876 and 19,469, respectively [9].

Based on the findings of previous studies, structural, financial, personal, and cultural factors are the most common barriers to HSU among PLHIV [2]. In two recent quantitative studies conducted in Iran, Jaffari et al. examined health service utilization among PLHIV in Kerman City and highlighted facilitators including trusting in the health system, having supportive family attitudes, benefiting from convenient service hours, and being aware of consultation benefits; meanwhile, identified barriers included financial constraints, transportation limitations, stigma, concerns about service quality, and lack of family support [10]. In parallel, Ghasemi et al’s qualitative investigation, which focuses on Afghan immigrants, reveals themes impacting access to HIV services, encompassing cultural considerations, psychosocial factors, and service delivery challenges [11]. Other barriers to HSU include the fear of HIV-related stigma and discrimination [12–14], fear of HIV status disclosure [15, 16], long waiting time at health centers [17], issues related to privacy and confidentiality [14, 17], staff shortage, poor relationship between patients and healthcare providers, incorrect and inadequate knowledge [16], lack of awareness about available interventions [18], geographical distance from healthcare centers, high healthcare costs, negative attitudes of healthcare providers, and negative religious beliefs [17]; which have been widely documented as major barriers of health services utilization by PLHIV.

According to the evidence, even minimal barriers are enough to discourage PLHIV from seeking and utilizing health services [19]. Existing studies suggest that PLHIV, who are aware of their status, may not seek healthcare services because they are unaware of available treatment and care options, or have poor access to health care services due to HIV-related stigma. Denial of HIV status may also be another barrier, in addition to lack of financial support [1, 2, 20, 21]. Non-utilization or under-utilization of health services not only affects the well-being of PLHIV but also poses a complex challenge to the healthcare system [22].

To address this issue and effectively plan for service provision, comprehensive data on the factors influencing HSU among PLHIV is essential. To the best of our knowledge, very little is known about the experiences, determinants, and obstacles to health service utilization among Iranian PLHIV. Thus, this study was conducted to address this gap in the literature by exploring health service utilization and also identifying the determinants and obstacles to HSU among PLHIV in Tehran, Iran. The findings of this study have implications for health policymakers to design and implement appropriate and targeted interventions that could be effective in improving health service utilization among PLHIV.

## Methods

### Study design

This is a qualitative study using conventional content analysis. The use of a qualitative approach in this research can be justified from several perspectives. The existing questionnaires for assessing the well-being of elderly living with HIV cannot capture all aspects of the subject. In this regard, having a qualitative approach and providing autonomy and sufficient time for the interviewees to express their perceptions and hidden aspects, which may be missed by the questionnaire, are important. Additionally, the objective of this study is to understand and comprehend a phenomenon for planning and policy making purposes, therefore, a qualitative approach is more suitable than a quantitative approach. In this study, data were collected using purposive and theoretical sampling, as well as semi-structured telephone interviews with PLHIV. The data were analyzed by the method suggested by Graneheim and Lundman [23, 24].

### Participants

The study included individuals aged 18–64 living with HIV in Tehran. The inclusion criteria consisted of a documented history of prior referrals to the behavioral counseling centers associated with Iran University of Medical Sciences, recent use of at least one health service within the last six months, having an interest to participate in the study, having the ability to communicate with the interviewer, and giving verbal informed consent. Exclusion criteria included leaving or not willing to participate in the interview, having a mental disorder, and having comorbidities which limit participation.

### Data collection

In this study, purposeful and theoretical sampling methods were incorporated (Lingard et al., 2015). Purposeful sampling was used to select the interviewees, and theoretical sampling was used to identify the number of participants, location of the required data, and the research path. This type of sampling with a variety of information, makes the nature and different aspects of the phenomenon more clear [23, 25]. Accordingly, by preparing a sampling matrix, 16 men and women aged 18–64 years living with HIV / AIDS in Tehran were selected based on age, gender, education, marital status, and employment, ensuring maximum variation. Data were collected after receiving the ethical code (IR.IUMS.REC.1398.822) from Iran University of Medical Sciences between September and December 2021. As a consequence of the COVID-19 pandemic, face-to-face interviews were not possible. After correspondence with the Infectious Diseases Deputy of the University and Behavioral Disease Counseling Centers affiliated with the Iran University of Medical Sciences (Valfajr, Taghinia, Khorshid, Shahriyar, Baharestan, and Robat Karim) and also receiving patients’ telephone numbers, the sample process and telephone interviews began. Data were collected by the first and third authors through semi-structured interviews using an interview guide. The interview guide and questions were designed and compiled in three sessions with the participation of the entire research team for this study and have not been used in other researches before. Also, to evaluate it, three test interviews were conducted and analyzed based on the designed questions, and after fixing some minor problems, the final version of the questions was used for the interviews (Table 1). The main focus of the questions was on the determinants of health service utilization by PLHIV. During the interview, the researchers first introduced themselves and briefly explained the objectives and necessity of the research to the participants, and after obtaining verbal informed consent, the interview began with several demographic questions, and then continued with the main questions.

**Table 1:** Interview guide

No	Question
1	Have you needed any health services in recent months (last 6 months)? Please explain.
2	Have you been referred to health centers to receive the required services? Please explain.
3	Have you received the required health services by being referred to the health centers? Please explain.
4	For what reasons were you referred to the health centers to receive the needed health services? What conditions motivated you to visit health centers? Please explain.
5	Are there any reasons that you didn’t go to the health center? What conditions prevented you from attending health centers? Please explain.

Along with the questions in the interview guide, exploratory questions were also asked according to the participants’ responses. All interview questions were asked of all participants, but the order of the questions varied according to the participants’ responses and interview circumstances. The interview time was set by the participants, and all interviews were conducted in the afternoon at their request. The duration of each interview varied from 35 to 70 min, with an average of 45 min. Interviews, data collection, and data analysis continued until data saturation was reached. Data saturation is achieved when new code is not found through analysis. In this study, saturation was obtained after 14 interviews, but the researchers preferred to conduct two more interviews to ensure saturation. Finally, considering that no new code was obtained after the 16th interview, all interviews were analyzed and considered as sample size.

### Data analysis

Data management and analysis were conducted by the second and third authors using MAXQDA-2018, following the Granheim and Lundman method [24]. In the first step, the researcher typed the interviews verbatim immediately after each interview with the help of a colleague. In the second step, the interview text was read three times by the researchers very carefully to get a general understanding of the text. In the third step, all the interview texts were read line by line and word by word with precision, and the initial codes were extracted. In the fourth step, the codes that were similar in meaning and concept were classified in a category. The codes and categories were then placed into the main classes, which were more conceptually comprehensive and abstract, forming the main themes. Finally, in a joint meeting, the entire data analysis process was shared with the research team members, and their opinions were considered and applied.

### Trustworthiness

Guba and Lincoln’s criteria were used to improve the quality of results [26]. To increase the credibility of the research, the researchers employed maximum variation sampling selecting participants with diverse demographic characteristics like age and gender. At the end of each interview, the researcher’s general understanding of the interviewee’s statements was briefly shared with him/her for confirmation. At the end of the data coding and analysis, a table of categories, subcategories and codes with related quotes was provided to six participants to determine whether the researchers had reported their experiences correctly or not. To ensure confirmability, the researchers sent the analyzed data and obtained findings to four leading researchers in qualitative research and five physicians and health care providers who provide health services to PLHIV, and considered their opinions and comments while maintaining the originality of the results. Raw data and all field notes were also retained for future review. To increase the dependability of findings, all research colleagues were informed about data analysis and coding, and had opportunities to express their opinions during meetings.

Finally, the categories and sub-categories were given names with the approval of all authors. To increase transferability, a complete description of the research process, participants’ quotes, and the research findings were shared with five individuals who met the inclusion criteria but were not part of the study for approval.

### Ethical considerations

In order to adhere to the ethical principles, before conducting the telephone interview, verbal informed consent was obtained from the participants and the time of the telephone interview was arranged with them. All participants were told that participation in the study is voluntary and they could withdraw from the study at any time. They were also briefed about the interview process and how the results will be published. The principle of confidentiality was also explained to the participants.

## Results

This study was conducted among 16 PLHIV whose demographic characteristics are listed in Table 2. Also, two main themes, seven categories and 21 subcategories were formed from the data analysis (Table 3).

**Table 2:** Demographic characteristics of the participants

Participant	Gender	Age	Employment	Insurance	Education	Marital status	Number of children
1	Male	44	Plaster work	Yes	Secondary	Married	2
2	Male	45	-	No	Diploma	Married	1
3	Male	-	Office worker		BSc*	Single	-
4	Male	44	Chef	Yes	Diploma	divorced	-
5	Female	28	Unemployed	-	Primary	divorced	-
6	Female	45	Housewife	-		Married	3
7	Female	23	-	-	BSc	Single	-
8	Female	24	-	Yes	-	Married	2
9	Female	62	Housewife	No	Primary	Widow	3
10	Female	47	Artist	No	Diploma	divorced	2
11	Female	39	Seller	Yes	BSc	-	-
12	Male	46	Tailor	Yes	Secondary	Married	2
13	Male	56	Unemployed	No	Secondary	divorced	1
14	Female	49	Secretary	Yes	BSc	Married	3
15	Male	33	Driver	No	BSc	Single	-
16	Male	60	Driver		-	-	3

* Bachelor of Sciences

**Table 3:** Categories and subcategories related to the determinants of health service utilization

Main themes	Categories	Subcategories
Facilitators of health service utilization	Positive personality traits	Self-esteem, Resilience, acceptance of the disease
	Social factors	Appropriate socio-economic status, social support, existence of charities providing services
	Structural-behavioral determinants	Proper rules and supervision, honest behavior of staff
Inhibitors of health service utilization	Personal conditions	Undesirable personality traits, lack of need for treatment, having several diseases at the same time, High cost of treatment in the private sector, low life expectancy
	Insufficient knowledge of the disease	Inadequate knowledge and lack of information about illness, remorse and misconceptions, self-medication
	Negative consequences of disease disclosure	Fear of disease disclosure, stigma and social judgment, social exclusion and isolation, lack of privacy
	Difficult access to services	Lack of comprehensive and specialized centers, inadequate access and scheduling, improper behavior of service providers

### Facilitators of health service utilization

The first main theme refers to the factors and conditions that facilitate the process of health service utilization.

#### Positive personality traits

The first category is related to the characteristics of the individuals that motivate them to seek health services. These traits include self-esteem and acceptance of the disease.

##### Self-esteem

Participants stated that because of their self-esteem and sense of self-worth, they do not hesitate to disclose their illness in health care centers and try to receive appropriate services.

“I am a human being and have dignity. Although I am sick, I respect myself and I do not want to be ashamed. In times of need, I will definitely go to see my physician.” (Participant 9).

“Since I have tried to strengthen my self-esteem and also since I am not ashamed, I go to the health center with more ease and express my problems.” (Participant 15).

##### Resilience

Resilience is another facilitator of health service utilization. People develop a greater sense of patience and tolerance during the disease. They are often more accepting of the challenges and shortcomings. They have faced and overcome numerous challenges throughout their disease, which has built their resilience and inner strength.

“Ever since I found out I had this problem; I’ve been bracing myself for other problems. I have faced many challenges in these few years and I have overcome them all.” (Participant 11).

##### Acceptance of the disease

One of the most important factors facilitating the service utilization was the acceptance of the disease according to the patients. People, who had come to terms with their illness and considered it like any other disease, were more likely to receive services. These people, after coping with their diseases tried to see the world differently, and with the hope that they will survive, sought treatment.

“Honestly, the more I look around, the more I see people with this disease; one with more severe disease and one with less one. Like all, I say that I am sick and have to be treated. I no longer think about what others say because I am the most important person and I have to think about myself.” (Participant 14).

“I accepted that I have this disease and I need to get treatment to fight it. So I don’t care what other people think. I receive any medicine and service in this field.” (Participant 11).

#### Social factors

The second category found in this study included factors that have a social aspect such as appropriate socio-economic status, social support, and the existence of charities that provide service.

##### Appropriate socio-economic status

Participants stated that adequate financial resources made it easy for them to go to medical centers, even public and private health centers, and obtain the required drugs from the health centers or the black market. People with appropriate socio-economic status can even hire a private physician and nurse to provide care for them as required.

“If my social-economic status is not good, it will be more troublesome. I am in a good financial position now and have personal physicians, so I am relieved about this.” (Participant 14).

“… When you have money, believe me, your illness is not a problem. Among people like us, those who do not have money or appropriate social-economic status are definitely less likely to seek treatment. But if their financial conditions are good, they will also get treatment.” (Participant 7).

“If there is money, many problems will be solved on their own. When you have money, you can go to any doctor you like or get a home doctor. On the other hand, if you do not have a good financial situation, you have to take any medicine you are given, whether they are effective or not, but you have no choice.” (Participant 2)

##### Social support

Most of the study participants had not informed their relatives about their illness, but those who had informed first-degree members and friends were in a better mood and were more likely to receive health services. Informing the loved ones caused them to use their support and even the support of physicians and medical staff of behavioral counseling centers. This also helped them to feel that someone is with them when they are sick and even refer them to trusted physicians.

“When people around you care about you, guide you, empower you, not blame you and are by your side in sensitive situations, then you will try.” (Participant 11).

“… Everything gets easier when people support you, especially for us who are always afraid of being rejected. The support of friends and family and even the community makes it easier for us to go to the service centers and they even encourage us to seek treatment.” (Participant 3).

##### Existence of charities providing health services

A number of patients admitted that they receive services in charitable centers because they are free of charge and do not ask direct questions about their illnesses. They were more inclined to return to these centers in the future than public or private centers.

“Charitable centers are centering that one can trust. I wish they are given more power and authority because they are really compassionate and trustworthy.” (Participant 14).

“It is really much easier to receive treatment and services from centers that are well acquainted with our disease and provide for us any care without discrimination. Charities are among the centers that do this and it is easier to get services from them.” (Participant 9)

#### Structural-behavioral determinants

The third category found in this study is the structural-behavioral determinants that have an organizational aspect and show rules and reactions to patients.

##### Proper rules and supervision

The existence of special rules and regulations can lead to better utilization of services for people living with HIV, and vice versa. Service utilization will be greater if proper rules are set for this situation and at the same time there is sufficient and proper supervision over its implementation.

“If legal support and supervision are not good, people will not go for treatment and no one will dare to express their illness, but here and in recent years there are a series of laws that support. Supervision is also good and the conditions of receiving services are good.” (Participant 15).

“Because of legal protection and good supervision, all people would have gone for treatment and no one would have been afraid to express his/her illness…” (Participant 15).

##### Honest behavior of staff

Due to the special circumstances of these people, there is some pessimism and concern about how healthcare staff will react to them. Honest behavior and speech of health care staff and service providers towards these people will increase their trust and this in turn, will increase the utilization of services by them.

“Sometimes you go to these health centers that are near your home and you may not want others to know about your illness, but the employees follow this matter. If you go to one of these centers, you will realize that even though they know about your illness, they treat you honestly. This honesty and attitude make you want to use health services.” (Participant 11)

### Inhibitors of health service utilization

The second theme in this study included factors that prevented service utilization by PLHIV.

#### Personal conditions

The first obstacle to utilizing of health services is personal and living conditions, which determine the patient’s decision on health service utilization.

##### Undesirable personality traits

The undesirable personality trait of patients was one of the reasons for their reluctance to receive health services. These traits included frustration, laziness, shyness, stress, carelessness, and lack of confidence about comorbidities and the need to receive these services, which prevented them from seeking health services.

“… Honestly, some of us do not go to visit a doctor because of laziness. We are kind of careless, and we have reached a stage where we do not pay much attention to our illness.” (Participant 4).

“Many people living with HIV have personality problems and are shy. You think that everyone is looking at you and you don’t like to appear in public.” (Participant 8).

##### Lack of need for treatment

Lack of need for treatment was one of the barrios to health service utilization according to the participants. According to the participants, as soon as a sick person feels he is in good physical and mental condition, he does not feel the need to go to the necessary check-ups in middle and old age to receive treatment.

“Honestly, I have not felt the need to seek treatment so far, and I may have an illness, but I do not seek health services” (Participant 3).

“I obtain the necessary medications from the market and perfumeries, and I no longer pay for doctor visits. I do not have to go to a place where everyone looks at me. I do not need to go to such places at all.” (Participant 3).

##### Having several diseases at the same time

Having an underlying disease increases treatment costs. Accordingly, if people suffer from co-morbidities in addition to HIV, they would prefer to receive services related to their co-morbidities and refuse to receive services related to AIDS.

“I do not see many doctors and do not utilize health services due to various diseases that I have. I have sinusitis, lung problems, and breast problems.” (Participant 9).

“I have both diabetes and digestive problems, and they bother me so much. These conditions cost me so much that I no longer have the money to receive services related to my disease. They are a priority and no one blames me, and it is easier for me to visit doctors for these conditions.” (Participant 8).

##### High cost of treatment in the private sector

Some patients do not have health care insurance, and some services are not provided in hospitals or are time-consuming, and for some reason, these services are provided by the private sector. The price difference between the public and private sectors has made patients with low socio-economic status or generally low income receive these services less often, because, for the majority of patients, the price of medications, health services and doctor visits are expensive. They do not have the financial means to receive such services.

“Private sector is very expensive and I cannot afford it.” (Participant 6).

“If the costs were lower and patients were insured, people would have used these services in the private hospitals and libraries.” (Participant 12).

“The situation is really such that you can’t go to a health center even if you want to. Every once in a while I have to go for a test, and every time I have to spend 600 to 700 thousand Toomans in a private laboratory.” (Participant 3).

##### Low life expectancy

Some patients, after learning about the disease or given information about the disease, completely lose hope or have little hope of survival. Low life expectancy discourages these people and prevents them from seeking health services.

“When I know I’m not alive for a few more years, then why should I go to the doctor? I better get sick sooner and die.” (Participant 14).

“When I know I cannot live a few more years even if I receive the best services, what difference does it make what services I receive? So it is not worth it for me.” (Participant 4).

#### Insufficient knowledge and understanding of the disease

Misconceptions and lack of knowledge about HIV were other issues that challenged utilizing of health services according to the participants.

##### Inadequate knowledge and lack of accurate information about the disease

Insufficient knowledge of the disease and the reasons for its transmission, and also incomplete information of some health care providers are the reasons for these patients not using health services. Participants stated that the lack of sufficient information about the ways of disease transmission and prevention, and also the presumption of certain death after infection as well as resulting stigma were among the problems they face. The lack of knowledge causes healthcare providers to refuse to provide services to PLHIV as soon as they know they have HIV.

“Before I got this disease, I did not think that this disease can be controlled. In the community and at school, we had not been told anything about this disease. Even our doctors do not have adequate knowledge about it. They have the information and learned about it, but when they want to give us treatment, they are reluctant to do so. So, what can we expect from others? Although in recent years, people’s knowledge has increased a lot.” (Participant 6).

##### Remorse and false beliefs

A number of patients feel guilty about the disease and have some false beliefs about it. Even some members of the community and academics have the same beliefs. As a result, feeling of guilt and false belief are among the major barriers to health services utilization by PLHIV.

“Sometimes I get upset when I go for an injection. It’s true that health care staff do not treat me differently, but if God forbid they have a needle stick in their hand, I will blame myself for it. I ask God why me. I used to get depressed and scared that my grandchildren would get the disease from me, although I later found out that the disease is not transmitted so easily and now we eat at the same table together.” (Participant 8).

“One belief is that whoever is infected with this disease will die after a while, and whatever he does, he will die soon. So, it is not necessary to bother me so much.” (Participant 10).

##### Self-medication

A number of patients, for reasons such as believing in the effectiveness of self-medication and traditional medicines, do not see physicians for various diseases and prefer to treat themselves through traditional medicines and self-medication. Most of these self-medications include the use of medicinal herbs offered by perfumeries and, to some extent, arbitrary use of medications bought from pharmacies and even the black market.

“… Now there are a lot of medications in every house, in perfumeries and even in supermarkets. I try to self-medicate before having to go to the doctor, and I do not go to the doctor unless I really have to.” (Participant 10).

“My friend and I try to self-medicate and solve our problem. For example, we use cloves for toothache.“ (Participant 6).

“When I have a cold, I try to take a pill or remedies, especially if I have to visit a doctor other than my own doctor.” (Participant 8).

#### Negative consequences of disease disclosure

The third obstacle to health service utilization is the fear of disease disclosure and its consequences, which prevent patients from going to health centers.

##### Fear of the disease disclosure

Patients always fear that they are at risk of disease disclosure. They are afraid of being disposed to friends, relatives, and family members when they explain their disease to healthcare providers. As a result, they try to talk less about their disease and attend health centers less often. In many cases, patients hide their secrets and do not inform their illness to a treating physician, even in high-risk cases of the disease.

“Everywhere I go, I start praying not to be exposed. I say, God; do not disgrace me and help me not to die with disgrace.” (Participant 15).

“PLHIV try to hide their disease even from their family when they visit a doctor.” (Participant 7).

##### Stigma and social judgment

Participants acknowledged that they are viewed as guilty by the community and there is a negative view of this disease. They also said that they are sexually labeled by ordinary people and even medical staff when their illness is exposed. Women are viewed as prostitutes and men are viewed as lustful. It is assumed that if a woman or a man is infected with this disease, he/she must have had sexual intercourse or used an infected needle, tattoo, surgery, non-sterile dental equipment, and even hairdressing tools. People do not think about their actions and offend these patients.

“I don’t want to say I’m sick because they point fingers at me. They do not know that I got this disease from my husband. They think I had sex with others.“ (Participant 3).

“We cannot say in society that we are sick, because people stigmatize us. They think we got it through, for example, sex with strangers or drug use, etc.” (Participant 1).

##### Social exclusion and isolation

Patients are secretive due to the fear of disease disclosure by family members, friends, and colleagues, and also the loss of their job. Some participants stated that they were rejected by friends and the community after disclosure of their disease. They said they have been excluded from their circle of relationships and interactions.

“If I say I’m sick, it will be spread rapidly among friends, family, and relatives, because I live in a small neighborhood and everyone will know about it and try to stay away from me.” (Participant 5).

“Society does not accept patients. When I told my close friend of a long time about my disease, first he asked a lot of questions about how I got sick, and then he was shocked. He cut his relationship with me in a way that I do not see him anymore, even though he knows that I have not made any mistake. Nevertheless, he had bad ideas about it and left me.” (Participant 10).

##### Lack of privacy

Some people living with HIV in this study stated that one of the main reasons they do not want to receive health services is the lack of privacy and confidentiality in health care centers. For example, when the treatment staffs realize that they have the disease, they ask many unnecessary questions such as: When did you get infected? How did you get infected? Why did you get infected? These questions bother patients, so they try not to visit random health centers.

“Once I went to a dentist and I told him about my disease. This word spread around and before you know it everyone was pointing figure at me. Since then, I try not to go to health centers unless I really need to, and when I go, I do not disclose my disease to anyone and do not say that I have AIDS.” (Participant 3).

“He looked at me in the lab with a contemptuous look and asked many questions: ‘Why did you get infected?‘ When did you get infected? Why did you get infected? He asked so many questions even though my condition had nothing to do with him while the visitors were also looking. He behaved very strangely and I was offended.” (Participant 10).

#### Difficult access to health services

The last category related to the barriers of HSU that were found in this study were reasons such as insufficient access to health services and inappropriate environment, which reduced the level of health service utilization by PLHIV.

##### Lack of comprehensive and specialized health centers

Although PLHIV would like to be accepted as normal people by the community and be served normally in health care centers, the majority of participants stated that it is necessary to establish special centers for PLHIV so that, they would refer and visit the centers with less concern and worry. Behavioral service centers only offer routine testing and HIV-related medications.

“It is good to have centers and clinics for PLHIV but these centers are limited, they are not forced to go to other places. These centers are only in certain places and accessibility to these centers is not possible for everyone.” (Participant 3).

“Unfortunately, the center that provides service to us does not have a dentist. Although the center refers us to trusted doctors, those doctors rarely set an appointment to treat us.” (Participant 2).

##### Inadequate accessibility to health services

A number of patients had to travel to Tehran to receive some services because they were living on the outskirts of the city, where some specialized services did not exist. The distance between these areas and Tehran is so great that some patients limit their use of health services, and even if they attend a center in Tehran, they encounter many problems due to the center’s busy scheduling.

“You know, it will cost me a lot of money to go somewhere else, as there is no health center near my home. The long distance from my home to Tehran discourages me to go to health centers. It’s really hard for me and others like me to access the health services we need.” (Participant 12).

“Access to Tehran is very difficult and it takes a lot of time. The transport fare is also too much. You have to leave home in the morning just to get there at noon. Then, you have to take a taxi back home. It’s not worth it for me” (Participant 9).

##### Inappropriate behavior of health care providers

According to the participants, among the unpleasant experiences of PLHIV in health centers are improper attitudes, views, and behavior of medical staff that offend them and reduce their access to health services.

“I and most patients like me usually do not inform the doctors that we have the disease. We are afraid that they will treat us inappropriately or they will not give us the necessary service.” (Participant 5).

“When you go to the doctor and say I’m sick, he looks at you like an impure thing. Some service providers treat us like criminals and that’s why I prefer not to go to those centers for treatment.” (Participant 6).

“All over the world, efforts have been made to treat this disease like other diseases. But in our society, even the health care staff, when they understand that you have this disease, they no longer serve you and stay away from you as if it is transmitted through eyesight” (Participant 8).

## Discussion

People living with HIV are faced with various personal and social difficulties, among which access to health care services is a major challenge [27, 28]. According to their special conditions, previous studies showed that HSU among PLHIV is lower than the general population [4, 29]; which according to the results of our study, is affected by a wide range of factors; these factors can be classified into two main categories of facilitators and barriers.

Positive personality traits including self-esteem, resilience, and acceptance of the disease, social factors such as social support and socio-economic status, in addition to structural and behavioral factors such as proper facility structure and friendly behavior of staff are among the facilitators of HSU. On the other hand, negative personal traits such as negative personality, low socio-economic status, insufficient knowledge of the disease, negative consequences of information disclosure, and difficult access to health services are among the barriers to HSU.

In line with other studies [2, 30, 31], our results indicated that individual-level factors such as self-esteem, resilience, and acceptance of the disease can affect HSU, as people who blame themselves and have lower self-esteem are less likely to seek help. We found that People with higher resilience are resistant to problems and even possible stigmas and are more likely to seek treatment and use services [32, 33]. Acceptance of disease and being reconciled with the presence of the illness allows adaptation to the life with HIV and this makes them seek services more often.

We found that one of the main factors that improve the access to health services by PLHIV is social factors, which include family support, in addition to the support provided by the community and charity centers where health services are provided. The role of social factors has also been highlighted in other studies; as community support, sense of belonging and having a supervising family member can improve the health outcomes of PLHIV [34, 35]. Having appropriate socioeconomic status is also crucial in receiving healthcare. Jessica and colleagues (2019) highlighted that higher socioeconomic status provides access to what is otherwise unattainable for other PLHIV [36]. Also, policies and the structure of health centers are key components in HSU [37].

In contrast, we also found other factors that act as barriers to HSU. Personal conditions such as undesirable personality traits, lack of need for treatment, and having several comorbidities at the same time are among the most common barriers to HSU. The high cost of services, Lack of ability to pay for health services and the social stigma associated with HIV are other factors that prevent PLHIV to utilize health services [38]. Therefore, implementing strategies to facilitate healthcare financing for these patients is necessary. The use of charities and subsidies or national integrated services has proved to be effective in this regard [39, 40]. As shown in other studies, stigma negatively affects the quality of life and access to health services [41], adherence to treatment and HSU among PLHIV [42]; which could stem from the tendency to keep a secret from healthcare providers [43, 44]. These points were prominent in our study as many people mentioned that social stigma has reduced their quality of life and also hindered their access to health services directly and indirectly. This stigma can come from healthcare professionals or friends, relatives, or the general population. The results show that general health sectors mainly have a negative attitude towards PLHIV, which create many challenges for them. This is a common challenge [29], but integrating different cost-effective services [10] can help to solve this problem. Having “Triangular Clinics” is a great advance in integrating health services and reducing harm, and this has proved to be effective in Iran. These clinics offer treatment and counseling for HIV, substance use, and sexually transmitted diseases [30].

According to our study, there is a lack of comprehensive policy regarding the access to health services by PLHIV. Many reasons could contribute to this problem, including not having enough health centers, not providing integrated services or financial support, and lack of insurance coverage. As the Ministry of Health and Medical Education is the main body for HIV-related policy-making in Iran, collaboration with all the stakeholders seems necessary to overcome these problems [45].

Another barrier to HSU is the lack of knowledge about the disease and related medical conditions. There are many PLHIV who are not aware of their disease, its consequences, or treatment methods. Many of these people are targeted by misinformation [46–48]. This lack of knowledge or misinformation causes people to consider HIV as an incontrollable disease, and prevents them from treatment seeking and utilizing services.

Since HIV is considered to be a sensitive subject among the Iranian public, there were some limitations to conducting this study. First, one of the most important limitations of the present study was the selection of participants only from PLHIV referred to VCTs, and those who were not referred to these centers were not included in our study. Second, some of the participants were not eager to take part in the study and share their experiences. This problem was resolved by replacing them with other people. Third, due to the COVID-19 pandemic, conducting face-to-face interviews was not possible, so we conducted the interviews over the phone. Due to the challenges that the participants were facing in their lives, it was hard for them to reveal some of the private aspects of their life. This problem was resolved by creating a bond between them and interviewer, plus, assuring them that whatever they say will remain confidential.

## Conclusion

Many studies have shown that health services utilization among PLHIV is lower than the general population; as they face many challenges that negatively affect their access to and use of healthcare services. Some factors such as stigma, financial hardship, and lack of social support can hinder their access to healthcare services; on the other hand, personal growth, integrated health services, and social support would enhance their access.

Policymakers must adapt policies to engage with PLHIV and avoid poor health outcomes; for instance prioritizing patients in receiving health services may serve as a successful strategy. It is also, recommended that policymakers invest more and expand specific services for PLHIV, while simultaneously increase the public knowledge about HIV to eliminate or reduce the related social stigma. Although much progress has been made over the past decade in understanding the problems of PLHIV, considerable gaps exist today, and much work remains to be done in the future. Even with effective anti-retroviral therapy, social exclusion and stigma seem to increase. Therefore, further studies of the challenges of PLHIV are surely needed.

## Data Availability

“The datasets generated and/or analysed during the current study are available from Dr. Neda SoleimanvandiAzar on reasonable request.“

## Abbreviations

HIV: Human Immunodeficiency Virus
HSU: Health Service Utilization
AIDS: Acquired Immune Deficiency Syndrome
PLHIV: People living with HIV
UNAIDS: The Joint United Nations Program on HIV
